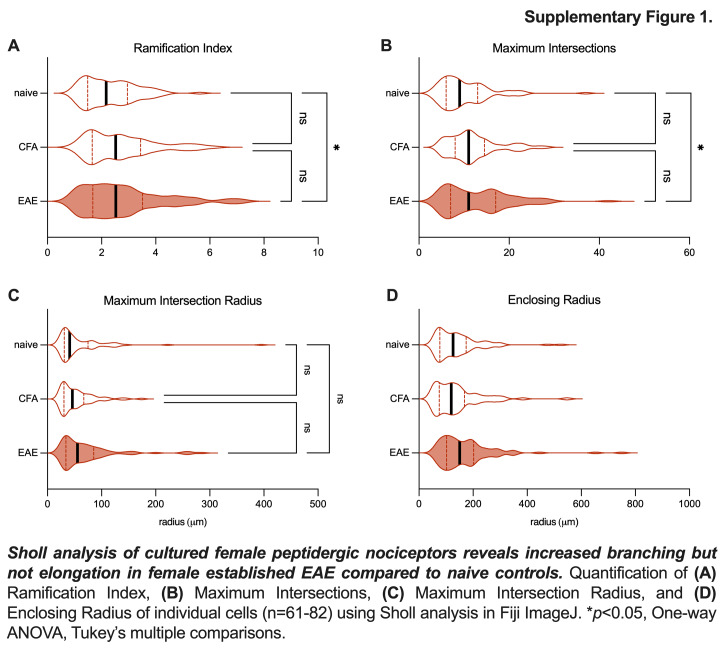# Sex-specific involvement of calcitonin gene–related peptide signaling for pain in experimental autoimmune encephalomyelitis

**DOI:** 10.1097/PR9.0000000000001350

**Published:** 2025-10-28

**Authors:** Aislinn D. Maguire, Timothy N. Friedman, Elyse Willis, Elise Gosse, Grayden Kuypers, Dania Villarreal Andrade, Camille Stephens, Gustavo Tenorio, Jason R. Plemel, Bradley J. Kerr

**Affiliations:** a Neuroscience and Mental Health Institute, University of Alberta, Edmonton, AB, Canada; b Department of Anesthesiology and Pain Medicine, University of Alberta, Edmonton, AB, Canada; c Division of Neurology, Department of Medicine, University of Alberta, Edmonton, AB, Canada; d Department of Medical Microbiology and Immunology, University of Alberta, Edmonton, AB, Canada; e Li Ka Shing Institute of Virology, University of Alberta, Edmonton, AB, Canada; f Department of Pharmacology, University of Alberta, Edmonton, AB, Canada

**Keywords:** Multiple sclerosis, Neuropathic pain, Plasticity, Peptidergic nociceptors, Sex differences

## Abstract

**Introduction:**

Neuropathic pain (NP) is one of the most devastating and under-managed symptoms of multiple sclerosis (MS). As it stands, NP is a difficult condition to treat, as many commonly used pain therapies are ineffective. Research has now emerged demonstrating sex differences in the mechanism of NP in MS, adding complexity to the search for new treatments. Although it is widely known that female patients are more likely than male patients to develop MS, it is less commonly acknowledged that they are also more likely to experience NP in the disease. Thus, there is an urgent need to develop NP treatments specifically for female patients with MS.

**Methods:**

Using the experimental autoimmune encephalomyelitis (EAE) mouse model of MS, we have characterized the outgrowth in culture as well as the spinal innervation pattern of peptidergic primary sensory afferents in both sexes. We then used a receptor antagonist to disrupt neuropeptide signaling from these cells to treat pain.

**Results:**

We found structural plasticity in peptidergic nociceptors from female animals with established EAE both in vitro and in vivo, as well as increased antibody stain intensity for the neuropeptide calcitonin gene–related peptide (CGRP). Using a receptor antagonist for CGRP (CGRP8-37), we were able to reverse spontaneous pain in female EAE animals.

**Conclusions:**

These results suggest that targeting peptidergic nociceptors and CGRP signaling, specifically in female patients, may be a viable strategy to relieve and possibly reverse pain in female patients with MS.

## Abstract 2

Supplemental Digital Content is Available in the Text.

Peptidergic neuron plasticity and signaling are potential targets to treat neuropathic pain in female patients with multiple sclerosis.

## 1. Introduction

Multiple sclerosis (MS) is a debilitating autoimmune disease characterized by cognitive, motor, and sensory symptoms. In MS, neuropathic pain (NP) is a particularly devastating symptom, which can manifest in both spontaneous and evoked modalities. Neuropathic pain in multiple sclerosis is extremely difficult to manage clinically due to its heterogeneous presentation and resistance to conventional pain treatments such as opioids.^18,25^ In addition, the treatments currently available have numerous unpleasant side effects.^2,23^ In MS, female patients are more likely than male patients (roughly 3:1) to develop the disease, and they are also more likely to experience NP (74.2% in female patients, 28.9% in male patients).^21,22^ Thus, there is an urgent need to develop new treatments for NP in MS with greater efficacy and sex specificity.

To address this need, we focused on the function of primary afferents. Although research into MS disease-modifying treatments focuses on the immune and central nervous system, the peripheral nervous system is a major contributor to neuropathic pain development.^12^ When peripheral nociceptors become sensitized due to inflammatory disease processes, their enhanced activity leads to ascending sensitization of pain pathways and ultimately NP.^1^ It is now hypothesized that once central sensitization is established, a cure for MS likely would not reverse NP. Indeed, in other autoimmune disorders, disease-modifying therapies fail to temper NP.^3^ Therefore, in this study, we focused on primary afferents and their connections in the spinal cord. We have previously found elevated peripheral immune cell infiltration, nociceptor hyperexcitability, and pain at disease onset in the experimental autoimmune encephalomyelitis (EAE) mouse model of MS.^13,17^ However, we have also found sex differences that suggest different mechanisms might underlie this pain in male patients and female patients. Immunohistochemistry revealed that more neurons in male dorsal root ganglia (DRG) neurons were stained with damage-associated proteins, suggestive of a neuropathy-like phenotype. However, female patients were stained with regeneration-associated proteins, suggestive of a plasticity-like phenotype.^13^ In this study, we sought to further explore this plasticity.

We found evidence for structural plasticity of primary peptidergic afferents both in vitro and in vivo in the deep dorsal horn (DDH) of the spinal cord, as well as increased antibody stain intensity for the neuropeptide calcitonin gene–related peptide (CGRP) in the DRG. Calcitonin gene–related peptide is known to amplify pain signaling and contributes to the sensitization of second-order neurons in the spinal dorsal horn,^7^ and its modulation is known to be effective in female-specific pain conditions including chronic migraine and endometriosis.^5,20^ Using CGRP8-37, a receptor antagonist, we were able to reverse already-established spontaneous pain as well as brainstem and DDH neuronal activation in EAE. These findings are in line with our previous work, which suggests that female EAE DRG neurons are more plastic than those from male patients.^13^ We propose that targeting signaling and/or plasticity of peptidergic neurons could be a viable treatment avenue for NP in female patients with MS.

## 2. Materials and methods

### 2.1. Experimental autoimmune encephalomyelitis

Male and female C57BL/6 mice (12–14 weeks of age) were induced as previously described^6,29^ with subcutaneous injection of 50 μg myelin oligodendrocyte glycoprotein (MOG_35-55_) emulsified in complete Freund adjuvant (CFA), followed by intraperitoneal injection of 100 ng of pertussis toxin on the same day, and the following day. Control animals received the same treatment, aside from the MOG_35-55_, and are referred to henceforth as “CFA.” Animals were followed for up to 22 days postimmunization then euthanized with intraperitoneal sodium pentobarbital injection (1200 mg/kg) and perfused with 20 mL cold 0.9% saline. All animal experiments were performed according to the University of Alberta Health Sciences Animal Care and Use Committee (AUP0000274).

### 2.2. Experimental autoimmune encephalomyelitis dorsal root ganglia cultures

Dorsal root ganglia cultures were adapted from reference 14 and performed as previously described in references 6, 14. Roughly 40 thoracic and lumbar DRGs from 2 mice per group were collected and enzymatically digested with 2 mg/mL STEMxyme I (Worthington LS004106) and 0.04% DNAse (Worthington LS002007) for 1 hour at 37°C, followed by trituration and centrifugation to remove enzyme. The pellet was then resuspended and passed through a 70-μm filter and centrifuged through a 15% Bovine serum albumin (BSA) cushion to remove myelin debris. Finally, cells were plated on a glass 24-well plate coated with poly-d-lysine and cultured for 48 hours in low-serum media Dulbecco's modified eagle medium (DMEM), 1% heat-inactivated fetal bovine serum, penicillin/streptomycin, GlutaMax, and sodium pyruvate).

### 2.3. Immunocytochemistry

Immunocytochemistry was performed as previously described.^13^ Following 48 hours in culture, cells were fixed for 15 minutes with equal parts warm media and 8% Paraformaldehyde (PFA) in 0.1 M PB (4% PFA final). Cells were then washed 3x with phosphate buffered saline (PBS) and blocked for 30 minutes at room temperature in 10% normal donkey serum (NDS) in 0.2% triton X-100 in PBS (PBS_TX_). Primary antibodies, incubated overnight at 4°C, included rabbit β3 Tubulin (1:1000, Sigma-Aldrich, Oakville, ON, Canada T2200), goat CGRP (1:500, Invitrogen, ThermoFisher, Wlatham, MA, USA PA1-85250), IB4 (1:500, Sigma-Aldrich L2140), and chicken NF200 (1:1000, Invitrogen PA1-10002). Cells were washed 3x with PBS, incubated with secondary antibody (1:500, Jackson ImmunoResearch, West Grove, PA, USA) and DAPI (1:3000, Invitrogen D1306), then washed 3x. Wells were flooded with PBS for storage and 10x imaging with a high content screening ImageXpress microscope in a 6-by-6 grid with 10% overlap.

### 2.4. Immunohistochemistry

Fixed tissue was incubated at 4°C in 4% PFA overnight, followed by 2 days in 30% sucrose in 0.1 M PB. The tissue was then embedded and frozen in Tissue-Tek medium (Sakura 4583) before cryosectioning. Spinal cords (lumbar enlargement) were sectioned at 20 μm thickness and DRGs at 10 μm. Once mounted on slides, tissue was washed in PBS and PBS with 0.05% Tween-20 (PBS_Tw_) and blocked for 1 hour at room temperature in 10% NDS in PBS_TX_. Primary antibodies including rabbit CGRP (1:100, Invitrogen PA5-114929, used in the third figure), goat CGRP (1:500, Invitrogen PA1-85250, used in the fifth, seventh, and eighth figures), rabbit GAP43 (1:1000, Sigma-Aldrich AB5220), and rabbit c-FOS protein (c-FOS) (1:500, Cell Signaling 2250, New England Biolabs, Danvers, MA, USA) were incubated overnight at room temperature. Slides were washed 2x with PBS_Tw_ and once with PBS before incubation with secondary antibodies (1:200, Jackson ImmunoResearch) and DAPI (1:1000, Invitrogen D1306). Slides were washed again before the excess liquid was tapped off and coverslips were mounted with 2 drops of mounting medium (Fluoromount-G, Invitrogen 00-4958-02).

### 2.5. Behavior and CGRP8-37 drug treatment

Spontaneous pain was assessed using PainFace software to measure grimace scale scores from 15-minute recordings.^16^ Baseline recordings were taken before EAE induction, which was then performed as described above. On day 15, when motor symptoms had onset, another recording was taken. This was followed by the administration of 2 μg per mouse daily intraperitoneal (IP) injections of CGRP8-37 in PBS to one EAE group and PBS alone to the other EAE and CFA control mice. The final recording was taken on day 22 *before* daily treatment with CGRP8-37. The mice then received their daily treatments as normal until 1 hour before euthanasia on day 23.

### 2.6. Data analysis and statistics

#### 2.6.1. Immunocytochemistry

Outgrowth and branching in the first figure were analyzed as previously described^6^ using the “Neurite Extension” plugin for MetaXpress 6. Data from EAE animals were expressed as fold change of either total mean outgrowth or total mean branches per cell, normalized to neurons from CFA vehicle control mice. Data points represent one well, n = 6 to 12 wells per condition with roughly 5 to 15 cells analyzed per image in a 6 × 6 grid. Costained outgrowth data in the second figure were expressed as fold change of mean outgrowth per cell normalized to naïve control mice. Data points represent one well, n = 4 wells per condition with roughly 6 to 29 cells analyzed per well. The mean outgrowth per cell (length) was analyzed using Sholl analysis from Fiji ImageJ. All data were analyzed by 2-way ANOVA with Tukey multiple comparisons. Any outliers were removed using the ROUT method.

#### 2.6.2. Immunohistochemistry

Calcitonin gene–related peptide *staining* data from male and female established EAE in the third figure (n = 6–8), and female drug experiment in the seventh and eighth figures (n = 5) in lumbar spinal cords was expressed as fold change of % stained area normalized to naïve controls. Segmentation between upper and deeper laminae was drawn by hand around the stained area. Data points represent a summary from 2 to 4 sections per animal. *GAP43 staining* data from male-established and female-established EAE lumbar spinal cords were expressed as fold change of % stained area normalized to naïve controls. Segmentation between upper and deeper laminae was drawn by hand and determined using CGRP stained area. Data points represent a summary from 2 sections per animal, n = 3 to 4 animals per condition. *CGRP intensity* data from female established EAE DRGs were expressed as fold change of mean gray value normalized to naïve controls. Calcitonin gene–related peptide–positive cells were expressed as fold change of CGRP-antibody–stained cells/mm^2^ and CGRP-antibody–stained DRG somas >30 μm over total CGRP-antibody–stained cells, both normalized to naïve controls. Data points represent one section per animal, n = 5 to 6 animals per condition. *c-FOS staining* data from female drug experiment in the lumbar spinal cord dorsal horn and the Sp5 level of the trigeminal nucleus of the brainstem were expressed as fold change of positively stained cells in the field of view (which was selected based on CGRP staining). Data points represent 1 to 2 sections per one animal, n = 5 animals per condition. All analyses were performed using Fiji ImageJ. Data were analyzed by 2-way ANOVA with Tukey multiple comparisons. Any outliers were removed using the ROUT method.

#### 2.6.3. Experimental autoimmune encephalomyelitis scoring and grimace

Experimental autoimmune encephalomyelitis scores were expressed out of 4, where 1 represents a limp tail, 2 includes unilateral hindlimb paralysis, 3 includes full hindlimb paralysis, and 4 represents quadriplegia.^8^ Data points represent the average per group, n = 5 animals. Data were analyzed by repeated measures 2-way ANOVA with Tukey multiple comparisons. No outliers were removed. *Grimace scores* were expressed as fold change of mean score over a 15-minute recording period. Data points represent the average per group, n = 5 animals. Analysis of facial grimace was performed using automated PainFace software.^16^ Data were analyzed by repeated measures 2-way ANOVA with Tukey multiple comparisons. One outlier was removed due to malocclusion possibly affecting pain and facial grimace behavior.

## 3. Results

### 3.1. Outgrowth is increased in female calcitonin gene–related peptide+ dorsal root ganglia neurons in vitro

**Figure 1.**
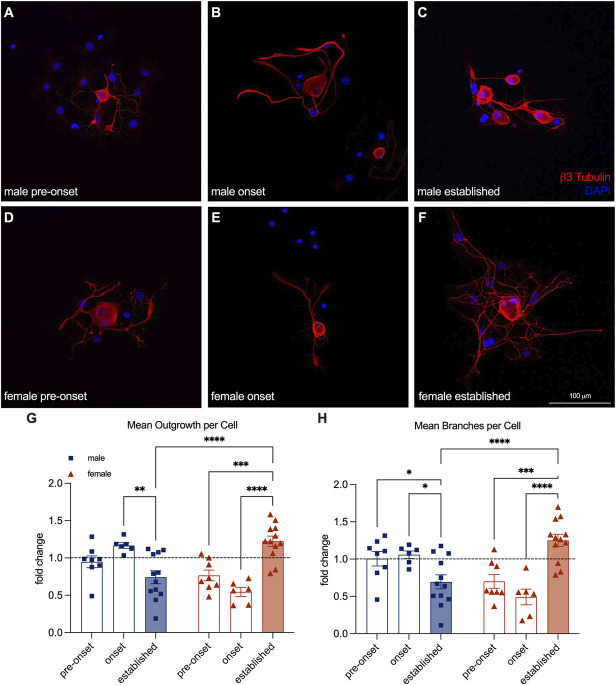
Outgrowth and branching are increased in sensory neurons from female animals with established EAE in vitro. (A–F) Representative images from cultures of male and female DRG neurons from preonset, onset, and established EAE mice stained with β3-tubulin to quantify neuronal outgrowth. (G) Quantification of mean outgrowth per cell expressed as fold change over experimentally matched wells from CFA control animals. (H) Quantification of mean branches per cell expressed as fold change over experimentally matched wells from CFA control animals. Bars represent mean ± SEM, n = 6 to 12 wells per group. *P < 0.05, **P < 0.01, ***P < 0.001, ****P < 0.0001, Tukey multiple comparisons test, 2-way ANOVA. DRG, dorsal root ganglia; CFA, complete Freund adjuvant; EAE, experimental autoimmune encephalomyelitis.

Previous work from our laboratory has indicated that DRG neurons from female mice with EAE express more histological markers indicative of neural plasticity, while male DRGs from diseased mice have more features of degeneration and neuropathy.^13^ In this study, we asked whether these histological signatures in situ translate into structural neural plasticity. We began by assessing the structure of EAE primary DRG neurons in vitro (stained β3-tubulin antibody) and analyzing neuronal outgrowth at 3 time points (Figs. 1A–F). The 3 time points assessed were preonset (∼10 days after induction, no motor symptoms), “onset” (∼14 days after induction, motor signs first becoming apparent), and “established” (∼21 days after immunization, motor symptoms are severe). Experimental autoimmune encephalomyelitis disease score was determined using a previously described 4-point scale.^8^ The most apparent difference occurred at the established disease time point, where male EAE neuronal outgrowth per cell decreased compared with the onset time point (onset mean = 1.17, established mean = 0.74 *P* = 0.0016, Tukey multiple comparisons test, 2-way ANOVA), and female EAE neuronal outgrowth increased in established compared with both preonset and onset (preonset mean = 0.77, onset mean = 0.55, established mean = 1.22, preonset vs established *P* = 0.0002, onset vs established *P* < 0.0001, Tukey multiple comparisons test, 2-way ANOVA). There was also a significant difference between the sexes at the established time point (male mean = 0.74, female mean = 1.22, *P* < 0.0001, Tukey multiple comparisons test, 2-way ANOVA) (Fig. 1G). The number of branches per cell followed the same trend where male EAE branching per cell decreased in the established time point (preonset mean = 1.00 onset mean = 1.06, established mean = 0.69, preonset vs established *P* = 0.044, onset vs established *P* = 0.029, Tukey multiple comparisons test, 2-way ANOVA), and female EAE neuronal branching increased in the established time point (preonset mean = 0.70, onset mean = 0.49, established mean = 1.25, preonset vs established *P* = 0.0002, onset vs established *P* < 0.0001, Tukey multiple comparisons test, 2-way ANOVA). There was significant difference between the sexes at the established time point (male mean = 0.69, female mean = 1.25, *P* < 0.0001, Tukey multiple comparisons test, 2-way ANOVA) (Fig. 1H). These results support our theory that female mice display plasticity-like phenotype, and male mice exhibit a neuropathy/degenerative-like phenotype.

**Figure 2.**
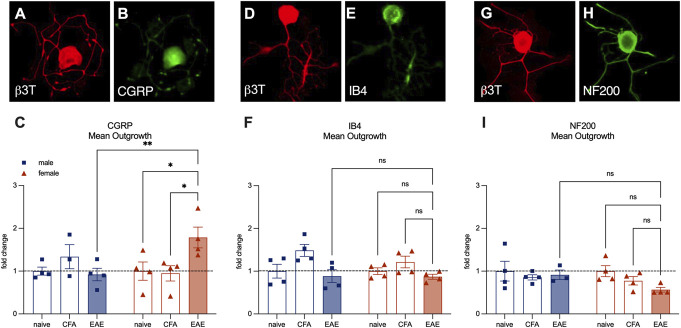
CGRP-antibody–stained peptidergic afferents are responsible for increased outgrowth from female sensory neurons from established EAE in vitro. (A and B) Representative images a female-established EAE neuron stained with a CGRP to identify peptidergic nociceptors and with β3-tubulin to quantify outgrowth. (C) Quantification of mean outgrowth per cell expressed as fold change over naïve control animals. (D and E) Representative images of a female-established EAE neuron stained with an IB4 antibody to identify nonpeptidergic nociceptors and with β3-tubulin to quantify outgrowth. (F) Quantification of mean outgrowth per cell expressed as fold change over naïve control animals. (G and H) Representative images of a female-established EAE neuron stained with an NF200 antibody to identify large diameter myelinated afferents and with β3-tubulin to quantify outgrowth. (I) Quantification of mean outgrowth per cell expressed as fold change over naïve control animals. Bars represent mean ± SEM, n = 4 wells per group. *P < 0.05, **P < 0.01 Tukey multiple comparisons test, 2-way ANOVA. CGRP, calcitonin gene–related peptide; EAE, experimental autoimmune encephalomyelitis.

Next, we costained cultured cells from the established time point to identify 3 subpopulations of DRG neurons. We used a CGRP antibody to identify peptidergic nociceptors (Figs. 2A–C), an IB4 antibody to identify nonpeptidergic nociceptors (Figs. 2D–F), and an NF200 antibody to identify large diameter myelinated afferents (Figs. 2G–I). Outgrowth from female EAE CGRP+ nociceptors was increased (Fig. 2C) (female naïve mean = 1.00, female CFA mean = 1.10, female EAE mean = 1.79, male EAE mean = 0.92. female naïve vs EAE *P* = 0.025, female CFA vs EAE *P* = 0.017, male EAE vs female EAE *P* = 0.0052, Tukey multiple comparisons test, 2-way ANOVA). Sholl subanalysis revealed increased branching but not elongation of these neurons (Supplemental Figure 1, http://links.lww.com/PR9/A351). IB4+ neurons (Fig. 2F) and NF200+ neurons (Fig. 2I) exhibited no statistically significant changes. These results demonstrate that female-established EAE peptidergic nociceptors exhibit heightened structural plasticity in vitro.

### 3.2. Calcitonin gene–related peptide antibody staining is increased in the deep dorsal horn of females with established experimental autoimmune encephalomyelitis

**Figure 3.**
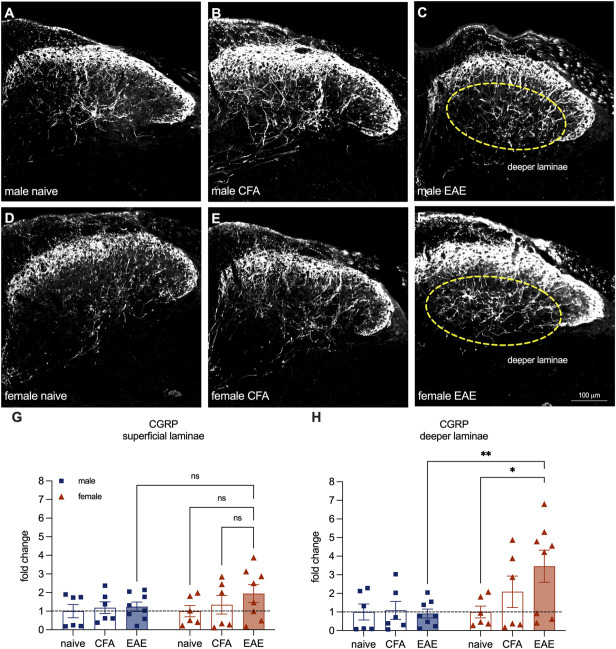
CGRP-antibody–stained peptidergic afferents are increased in the deeper laminae of the lumbar spinal dorsal horn in female animals with established EAE. (A–F) Representative images of CGRP antibody staining in the spinal dorsal horn from male and female naïve, CFA control, and established EAE animals. (G) Quantification of % CGRP-antibody–stained area in the upper laminae of the spinal dorsal horn expressed as fold change over naïve controls. (H) Quantification of % CGRP-antibody–stained area in the deeper laminae of the spinal dorsal horn expressed as fold change over naïve controls. Bars represent mean ± SEM, n = 6 to 8 animals per group. *P < 0.05, **P < 0.01 Sidak multiple comparisons test, 2-way ANOVA. CFA, complete Freund adjuvant; CGRP, calcitonin gene–related peptide; EAE, experimental autoimmune encephalomyelitis.

To determine whether the structural plasticity in EAE occurs in the spinal cord, we stained lumbar spinal cords with a CGRP antibody (Figs. 3A–F). Primary CGRP+ afferents synapse primarily in the upper laminae of the DH (lamina I and II outer)^1^ and in the deeper DH (lamina V).^7^ Therefore, we segregated the DH into superficial vs deeper laminae for analysis. In the superficial laminae, we saw no significant differences in the percentage of CGRP-antibody–stained area (Fig. 3G). However, in the deeper laminae, we found a greater stained area in female EAE cords (female naïve mean = 1.00, female EAE mean = 3.46, male EAE mean = 0.92. female naïve vs EAE *P* = 0.016, male EAE vs female EAE *P* = 0.0024, Tukey multiple comparisons test, 2-way ANOVA) (Fig. 3H). These results are in line with our in vitro findings, suggesting that peptidergic fibers are sprouting in female EAE animals.

### 3.3. Dorsal root ganglia from female established experimental autoimmune encephalomyelitis have increased calcitonin gene–related peptide antibody staining intensity and do not reveal increased calcitonin gene–related peptide+ cells or evidence of phenotypic switching

**Figure 4.**
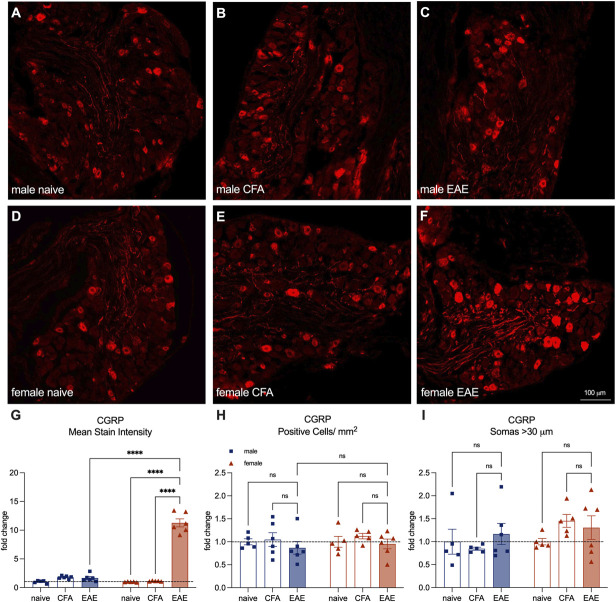
CGRP-antibody stain intensity increases in the DRG of female-established EAE animals, but staining in large diameter cell bodies does not increase. (A–F) Representative images of CGRP antibody staining in male and female naïve, CFA, and established EAE. (G) Quantification of CGRP mean stain intensity in the DRG expressed as fold change over naïve controls. (H) Quantification of CGRP-antibody–stained cells per mm^2^ of DRG cell body area expressed as fold change over naïve controls. (I) Quantification of CGRP-antibody–stained cells >30 μm over total CGRP-antibody–stained cells expressed as fold change over naïve controls. Bars represent mean ± SEM, n = 5 to 6 animals per group. Tukey multiple comparisons test, 2-way ANOVA. CFA, complete Freund adjuvant; CGRP, calcitonin gene–related peptide; DRG, dorsal root ganglia; EAE, experimental autoimmune encephalomyelitis.

We also assessed whether there were changes in CGRP staining in the DRG of EAE animals (Figs. 4A–F). First, we assessed mean gray value and found that female mice with established EAE had increased CGRP-antibody staining intensity (female naïve mean = 1.00, female CFA mean = 1.15, female EAE mean = 11.28, male naïve mean = 1.00, male CFA mean = 1.80, male EAE mean = 1.67. female naïve vs EAE *P* < 0.0001, female CFA vs EAE *P* < 0.0001, male naïve vs EAE *P* = 0.37, male CFA vs EAE *P* = 0.96, male EAE vs female EAE *P* < 0.0001, Tukey multiple comparisons test, 2-way ANOVA) (Fig. 4G). These results indicate that female EAE DRGs may be producing more CGRP.

We also assessed the number of cell bodies stained with the CGRP antibody. Phenotypic switching is a phenomenon described in mice after axotomy, wherein Aβ myelinated large diameter afferents begin to express neuropeptides such as CGRP, contributing to neuropathic pain.^19^ To determine whether this mechanism could be at play in EAE, we measured the proportion of DRG neurons expressing CGRP and found no changes (Fig. 4H). Furthermore, we measured the diameter of the DRG cell bodies (nociceptors are generally less than 30 μm in diameter, and Aβ cells are generally greater than 30 μm in diameter).^26^ When we analyzed cell bodies >30 μm, we still did not see a significant increase in the proportion expressing CGRP (Fig. 4I). These results suggest that phenotypic switching of Aβ afferents is not likely the cause of the increased CGRP+ neuron staining in the spinal cord of female EAE animals.

### 3.4. Increased GAP43 staining in the spinal dorsal horn indicates neuronal outgrowth and remodeling in female mice with experimental autoimmune encephalomyelitis

**Figure 5.**
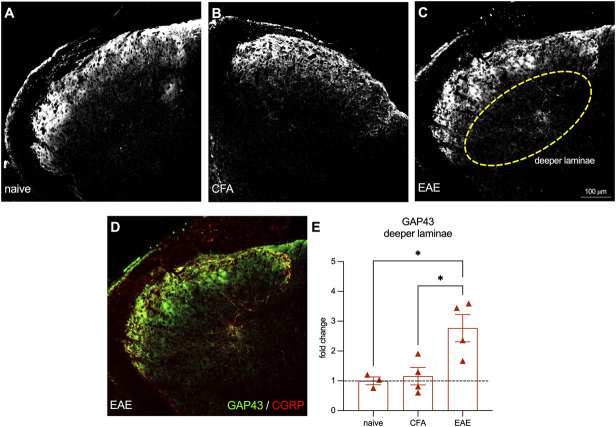
GAP43 antibody staining is increased in the deeper laminae of the spinal dorsal horn of female-established EAE animals. (A–C) Representative images of GAP43 antibody staining from female naïve, CFA, and established EAE animals. (D) Representative staining of GAP43 and CGRP antibody staining. (E) Quantification of GAP43% stained area in the deeper laminae of the lumbar spinal dorsal horn. Bars represent mean ± SEM, n = 3 to 4 animals per group. *P < 0.05, Tukey multiple comparisons test, 1-way ANOVA. CFA, complete Freund adjuvant; CGRP, calcitonin gene–related peptide; EAE, experimental autoimmune encephalomyelitis.

To support our theory that increased CGRP staining in the spinal DDH in female mice with EAE is due to structural plasticity, we stained the DDH of female animals with an antibody for GAP43 (Figs. 5A–C), as GAP43 has been linked to neuronal sprouting and neuropathic pain in animal models.^27^ We found that the GAP43-antibody–stained area in the DDH of female established EAE mice is increased (female naïve mean = 1.00, female CFA mean = 1.16, female EAE mean = 2.77, female naïve vs EAE, *P* = 0.022, female CFA vs EAE, *P* = 0.023, Tukey multiple comparisons test, 1-way ANOVA) (Fig. 5D). This finding provides evidence that peptidergic neurons in female mice with EAE may be sprouting and undergoing structural remodeling.

### 3.5. Treatment of experimental autoimmune encephalomyelitis mice displaying spontaneous pain with a calcitonin gene–related peptide receptor antagonist reverses pain and neuronal activation

**Figure 6.**
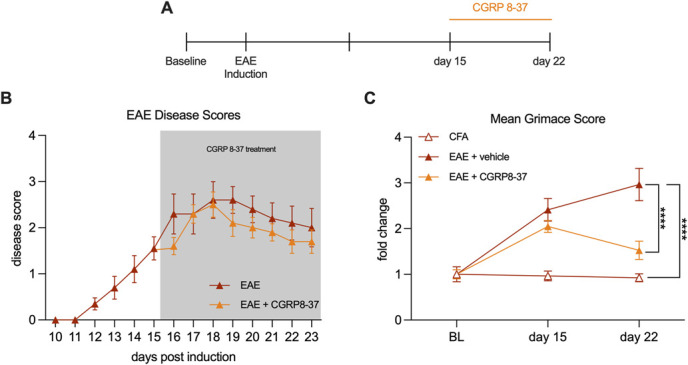
Treatment with the receptor antagonist CGRP8-37 reverses spontaneous pain in female animals with established EAE. (A) Timeline of EAE induction and CGRP8-37 (Beginning after grimace measurement on day 15. Day 22 measurement was taken before daily treatment). (B) EAE disease scores (out of 4) in EAE vehicle–treated and EAE drug–treated animals over the experimental time-course. (C) Mean facial grimace score of CFA, EAE vehicle, and EAE drug–treated animals at baseline, day 15 (before drug treatment), and day 22 (after daily drug treatment, but before treatment that day). Data points represent mean ± SEM, n = 5 animals per group. ****P < 0.0001, Tukey multiple comparisons test, repeated measures 2-way ANOVA. CFA, complete Freund adjuvant; CGRP, calcitonin gene–related peptide; EAE, experimental autoimmune encephalomyelitis.

Finally, we sought to determine whether interfering with peptidergic signaling through CGRP could reduce pain in EAE. We inhibited CGRP signaling with the receptor antagonist CGRP8-37 as it is well-tolerated and effective in rats and mice.^4,24^ Spontaneous pain was assessed using automated PainFace software which detects facial grimace. This allows for testing when EAE motor dysfunction and paralysis prevent reflex-based testing. Two groups of female mice were induced with EAE, alongside one CFA vehicle control group. On day 15, after disease onset, we began treating one group of EAE animals with 2 μg/d CGRP8-37 by IP injection and the other 2 groups with PBS (Fig. 6A). There were no significant differences in the disease scores of the animals after the initiation of treatment (Fig. 6B). We found that both EAE groups had increased mean grimace scores at day 15, before the initiation of treatment with CGRP8-37 (CFA mean = 0.97, EAE mean = 2.41, EAE + drug mean = 2.05, CFA vs EAE *P* < 0.0001, CFA vs EAE + drug *P* = 0.0011, Tukey multiple comparisons test, repeated measures 2-way ANOVA). On day 22, after 1 week of CGRP8-37 daily treatments, the EAE-vehicle group exhibited a higher grimace score than CFA controls, and the CGRP8-37-treated EAE group had returned to the level of CFA controls (CFA mean = 0.92, EAE mean = 2.97, EAE + drug mean = 1.53; CFA vs EAE *P* < 0.0001; CFA vs EAE + drug *P* = 0.088, EAE vs EAE + drug *P* < 0.0001; Tukey multiple comparisons test, repeated measures 2-way ANOVA) (Fig. 6C). These results suggest that treatment with CGRP8-37 reduced spontaneous pain in female EAE mice. Notably, PainFace measurements were taken before the daily injection with CGRP8-37, indicating these daily treatments may have a lasting and/or cumulative effect on pain.

**Figure 7.**
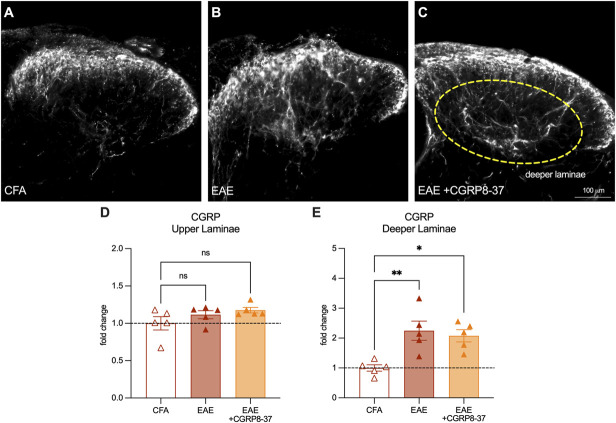
CGRP-antibody–stained peptidergic afferents are increased in the deeper laminae of the lumbar spinal dorsal horn in female animals with established EAE with or without CGRP8-37 drug treatment. (A–C) Representative images of CGRP antibody staining in the spinal dorsal horn from female CFA control, EAE vehicle control, and EAE drug–treated animals. (D) Quantification of % CGRP-antibody–stained area in the upper laminae of the spinal dorsal horn expressed as fold change over naïve controls. (E) Quantification of % CGRP-antibody–stained area in the deeper laminae of the spinal dorsal horn expressed as fold change over naïve controls. Bars represent mean ± SEM, n = 5 animals per group. *P < 0.05, **P < 0.01 Tukey multiple comparisons test, 2-way ANOVA. CFA, complete Freund adjuvant; CGRP, calcitonin gene–related peptide; EAE, experimental autoimmune encephalomyelitis.

**Figure 8.**
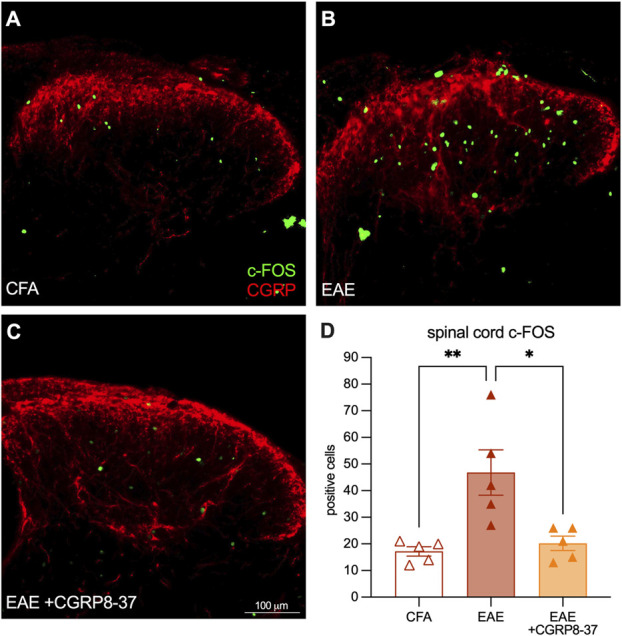
Increased c-FOS staining is ameliorated in the lumbar spinal dorsal horn in female established EAE. (A–C) Representative images of CGRP and c-FOS antibody staining in the lumbar spinal dorsal horn of female CFA, EAE vehicle, and EAE drug–treated animals representative images of CGRP and c-FOS antibody staining in the CGRP-stained area are of the trigeminal nucleus of the brainstem of female CFA, EAE vehicle, and EAE drug–treated animals. (D) Quantification of c-FOS-antibody–stained cells in the lumbar spinal dorsal horn expressed as fold change over CFA controls quantification of c-FOS-antibody–stained cells in the trigeminal nucleus of the brainstem expressed as fold change over CFA controls. Bars represent mean ± SEM, n = 5 animals per group. *P < 0.05, **P < 0.01 Tukey multiple comparisons test, 2-way ANOVA. CFA, complete Freund adjuvant; CGRP, calcitonin gene–related peptide; EAE, experimental autoimmune encephalomyelitis.

After euthanasia and tissue collection, we stained both the spinal cords and brainstems of these mice with a CGRP antibody to determine whether CGRP8-37 treatment influenced the plasticity of peptidergic fibers (Figs. 7A–C). We found no changes in the upper laminae (Fig. 7D). However, in the deeper laminae, we found that both the EAE vehicle and EAE drug–treated mice both had increased CGRP-antibody–stained fibers suggesting no effect of the drug on this metric (CFA mean = 1.00, EAE mean = 2.25, EAE + drug mean = 2.08, CFA vs EAE *P* = 0.0058, CFA vs EAE + drug *P* = 0.015, EAE vs EAE + drug *P* = 0.86. Tukey multiple comparisons, 1-way ANOVA) (Fig. 7E). We then turned our attention to postsynaptic neuron activity using a c-FOS antibody costained with CGRP to identify the area to quantitate. On the final experiment day, animals were given final IP injections one hour before euthanasia as c-FOS protein expression has been found to be most activated at this time point.^9^ We assessed the dorsal horn of the lumbar spinal cord (Figs. 8A–C) and the Sp5 trigeminal nucleus of the brainstem (as we used a facial readout of spontaneous pain) (Figs. 8D–F). In the spinal cord, the EAE-vehicle group had higher c-FOS+ cells than the CFA group, and the EAE-CGRP8-37 group had returned to the level of the CFA controls (CFA mean = 17.20, EAE mean = 46.80, EAE + drug mean = 20.20, CFA vs EAE *P* = 0.0049, CFA vs EAE + drug *P* = 0.92, EAE vs EAE + drug *P* = 0.010. Tukey multiple comparisons, 1-way ANOVA) (Fig. 8G). These findings were confirmed in the trigeminal nucleus of the brainstem (CFA mean = 8.60, EAE mean = 25.60, EAE + drug mean = 9.75, CFA vs EAE *P* = 0.0016, CFA vs EAE + drug *P* = 0.95, EAE vs EAE + drug *P* = 0.0042. Tukey multiple comparisons, 1-way ANOVA). These results suggest that, as expected, CGRP receptor antagonist treatment does not affect peptidergic neuron plasticity and reverses pain by limiting postsynaptic neuronal activation.

## 4. Discussion

We have previously described the emergence of behavioral (mechanical allodynia) and electrophysiological indices of hyperexcitability in small diameter DRG neurons in both male and female mice at the onset of EAE.^17^ However, we have also described sex differences in protein expression in EAE DRGs and the response of male and female DRG neurons to proinflammatory cytokines.^13^ We developed a working theory that the DRGs from female mice with EAE are prone to a plasticity-like phenotype, and male mice to a degenerative-like phenotype, and that these processes underlie the pain associated with EAE in each sex. Given the clinical prevalence of pain in female mice with MS, we sought to further characterize sex differences in plasticity in the EAE model to gain insight into potential therapeutic avenues to treat pain in female mice with the disease.

We began in vitro by assessing 3 disease time points for changes in neuronal outgrowth and plasticity. We found that female DRG neurons exhibited more outgrowth and branching at the established time point, indicative of structural plasticity. In addition, when we costained these cells with antibodies for 3 subtypes of DRG neurons, we found that peptidergic nociceptors were responsible for the increased outgrowth we observed in female mice. However, the decrease seen in male mice was not obviously produced by one population. Next, we investigated peptidergic afferents in spinal cord tissue from mice with established EAE and found more CGRP-antibody–positive neuronal afferents in deeper laminae III-V. We also stained DRGs and found increased CGRP antibody staining intensity in EAE female mice, suggesting greater CGRP production. However, we found no changes in the proportion of stained cells with a soma diameter above 30 μm (representative of large diameter mechanosensitive afferents). These observations suggest that large diameter mechanosensitive afferents are not producing CGRP de novo and thus not phenotypically switching. We also stained with an antibody for the neuronal plasticity marker GAP43 in the spinal cord, which we found to be increased in the DDH in females with the disease, further supporting our hypothesis that structural plasticity is occurring in the spinal cord. However, we cannot rule out the possibility that CGRP-expressing interneurons in the dorsal horn may be plastic and contributing to the staining seen in spinal cord tissue.^11^

To demonstrate the functional relevance of increased peptidergic neurons toward pain in EAE, we inhibited CGRP signaling with the receptor antagonist, CGRP8-37. We induced EAE in female mice and measured their spontaneous pain using the facial grimace scale analyzed with PainFace software.^16^ We allowed pain and clinical signs of the disease to develop normally until day 15, at which point we found that all mice with EAE had increased facial grimace scores compared with CFA vehicle controls. We then administered daily intraperitoneal injections of a CGRP receptor antagonist (CGRP8-37) in one group of EAE mice and PBS vehicle injections in the other groups. On day 22 postimmunization, before the daily injection, we found that the treated EAE group had decreased grimace scores compared with their EAE-vehicle counterparts. This finding indicates not only that disrupting CGRP signaling in EAE animals can reverse pain, but that once-daily treatment with this drug provides potent relief lasting into the following day. Finally, tissue from these animals showed that the treatment did not alter the peptidergic afferents in the spinal cord, but it did decrease postsynaptic neuronal activation in the spinal cord and brainstem.

Future experiments will be required to address whether aberrant peptidergic afferent plasticity can be prevented in female EAE animals and whether targeting other neuropeptides can also treat pain. In addition, there are potential limitations that should be considered. CGRP is known to act on immune cells,^28^ which should be a consideration in MS. Fortunately, the CGRP monoclonal antibodies used clinically for migraine are thought to have little effect on the immune system.^10^ In addition, a small tracking study following individuals with chronic migraine and MS who use CGRP inhibitors has not detected any worsening of MS symptoms.^15^

Taken together, our findings suggest that signaling from peptidergic afferents with aberrant contributes to pain in female EAE mice. We propose that targeting peptidergic afferent plasticity and/or signaling through CGRP are potential targets to treat neuropathic pain in female MS patients.

## Disclosures

The authors have no conflict of interest to declare.

## Supplemental digital content

Supplemental digital content associated with this article can be found online at http://links.lww.com/PR9/A351.

**Figure:**